# Mechanical optimization of plate-screw interfaces in variable-angle locking systems: a parametric study

**DOI:** 10.3389/fbioe.2025.1681460

**Published:** 2025-10-30

**Authors:** Chen-Chiang Lin, Shun-Ping Wang, Kun-Jhih Lin, Ming-Hsien Hu

**Affiliations:** ^1^ Department of Orthopedics, National Taiwan University Hospital Yunlin Branch, Yunlin, Taiwan; ^2^ Department of Post-Baccalaureate Medicine, College of Medicine, National Chung Hsing University, Taichung, Taiwan; ^3^ Department of Orthopedics, Taichung Veterans General Hospital, Taichung, Taiwan; ^4^ Technology Translation Center for Medical Device, Chung Yuan Christian University, Taoyuan, Taiwan; ^5^ Department of Orthopedics, Show Chwan Memorial Hospital, Changhua, Taiwan

**Keywords:** variable angle locking, biomechanical study, angulation, bending moment, bone plate

## Abstract

**Introduction:**

Variable-angle locking systems offer increased surgical flexibility by permitting off-axis screw insertion, with some designs allowing angulation up to 15°. However, clinical challenges such as screw loosening and insufficient mechanical stability data persist. This study aims to evaluate the impact of different plate hole design parameters on the locking strength of plate-screw head interface under various screw insertion angles, providing insights to optimize VALS performance in fracture fixation.

**Methods:**

Variable-angle locking system with Point-Loading Thread-In mechanism was designed for investigation. Variations in locking hole design were evaluated based on slot size (1.3 mm vs. 1.5 mm), number of thread columns (three vs. four), and cone angle (20° vs. 22°). A total of eight distinct design groups were included for analysis. The screws, inserted at different inclinations (0°–15°), and locked at 0.8 Nm were subjected to a cantilever load-to-failure test. Ultimate bending moment at the screw-head interface and failure mode of the locking mechanism were determined.

**Results:**

The results exhibited a decrease in bending strength with increasing screw angulation in both three- and four-thread designs, with all groups exceeding 1 Nm at 0° but falling below 1 Nm at 15°. The highest bending moment was 1.46 Nm in the three-thread-column design at 0°, compared with 1.32 Nm in the four-thread-column design. At 15° screw angulation, the highest moments were 0.92 Nm and 0.90 Nm for the three-thread and four-thread designs, respectively. The plate hole with 1.3 mm slot, three thread column and 22° cone design showed higher bending moment than all other designs at each insertion angles.

**Discussion:**

Plate hole design—specifically slot size, thread number, and cone angle—significantly influences locking strength. The optimal design (3 slots, 1.3 mm, 22°) offers superior mechanical stability, guiding device optimization.

## 1 Introduction

The introduction of fixed-angle locking screws in the early 2000s (e.g., PC-FIX; Synthes) revolutionized fracture fixation by enabling constructs that maintain relative stability where absolute stability is difficult to achieve (Borgeaud et al., 2000). Traditional locking plates derive their strength from forming a single-beam construct through fixed-angle screw fixation. However, one major limitation of this system is the inability to adjust screw trajectory based on individual fracture patterns or anatomical variations (Konstantinidis et al., 2010). To overcome this, variable-angle locking plate systems (VALS) were developed, allowing variable screw angulation while maintaining angular stability. These designs aimed to offer the potential to optimize plate placement and screw orientation according to fracture anatomy without compromising mechanical rigidity.

Despite their theoretical advantages, the clinical adoption of variable-angle locking screws has been limited due to several technical and biomechanical concerns. Studies have demonstrated that variable-angle constructs may exhibit reduced load to failure, particularly when screws are inserted at extreme angles or depending on the implant design (Hebert-Davies et al., 2013; Mehling et al., 2013; Tidwell et al., 2016). Furthermore, clinical reports have documented cases of variable-angle locking screw loosening, even in low-load regions such as the distal radius (Foo et al., 2013; Fowler and Ilyas, 2013; Nishiwaki et al., 2021; Tank et al., 2016; Collinge et al., 2023; Schmidt et al., 2024). Fowler and Ilyas (Fowler and Ilyas, 2013) reported one case of screw loosening among 39 distal radius fractures treated with variable-angle volar locking plates, whereas two mechanical failures were observed in a cohort of 55 patients (Nishiwaki et al., 2021). In two retrospective cohort analyses (Tank et al., 2016; Collinge et al., 2023), higher failure rates of variable-angle locking screws were seen in 22% and 36% of cases, respectively. Given the increased cost and potential for reduced mechanical integrity, further investigation into the biomechanical performance of these implants is warranted.

**FIGURE 1:**
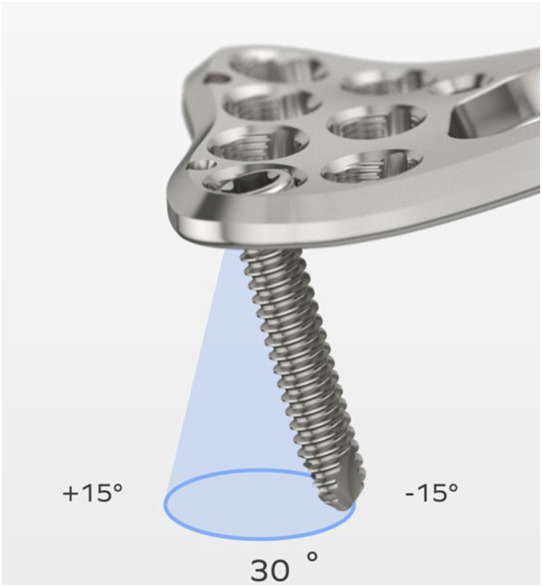
Example of a variable-angle locking plate with the point-loading thread-in mechanism.

The majority of mechanical studies focused either on comparing the locking strength of various variable-angle technologies (Hebert-Davies et al., 2013; Mehling et al., 2013; Tidwell et al., 2016; Foo et al., 2013) or on assessing the influence of multiple screw insertions on the failure load of a specific system (Glowacki et al., 2023a). However, comprehensive mechanical stability data for contemporary VALS remain unavailable, and the variety of currently VALS technology influence the biomechanical performance. Among FDA-cleared variable-angle designs, point-loading thread-in mechanisms—where screws engage lightly threaded tabs within the plate—are most frequently used clinically (Figure 1). By using multiple thread columns (formed by several slots), this technology achieves several locking points between the conical plate hole and the spherical screw head, enabling a fixed-angle construct with unrestricted screw trajectories (Chan and Fernandez, 2013). From a mechanical perspective, slot size, number of thread columns, and cone angle influence thread engagement, thereby affecting locking strength. This study aims to investigate the effect of design parameters of variable-angle locking plate holes on plate/screw locking strength at different screw angulations.

## 2 Materials and methods

### 2.1 Design parameters of the variable-angle locking hole

**FIGURE 2:**
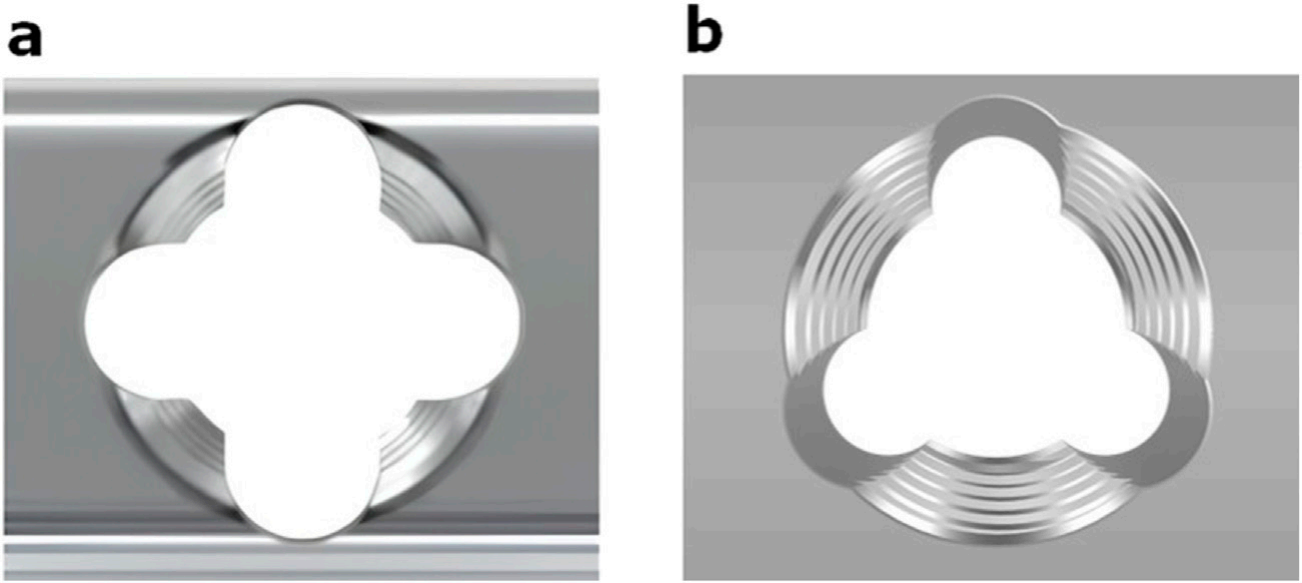
**(a)** Schematic of a variable-angle locking hole with four slots forming four columns of threads (DePuy Synthes). **(b)** Variable-angle locking hole with three slots (three thread columns).

**FIGURE 3:**
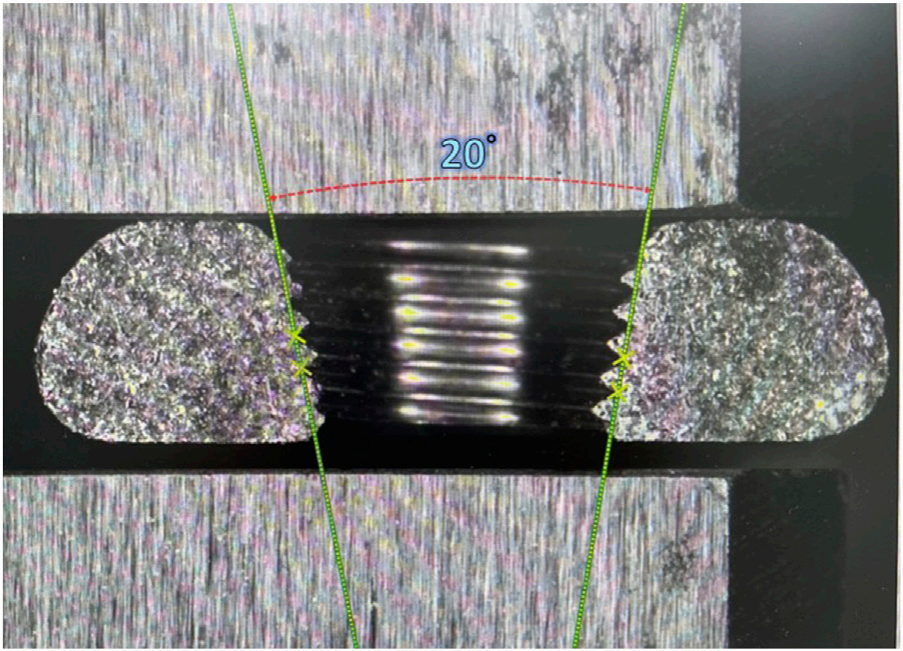
Cross-section of the generic locking plate hole showing a cone angle of 20°.

**FIGURE 4:**
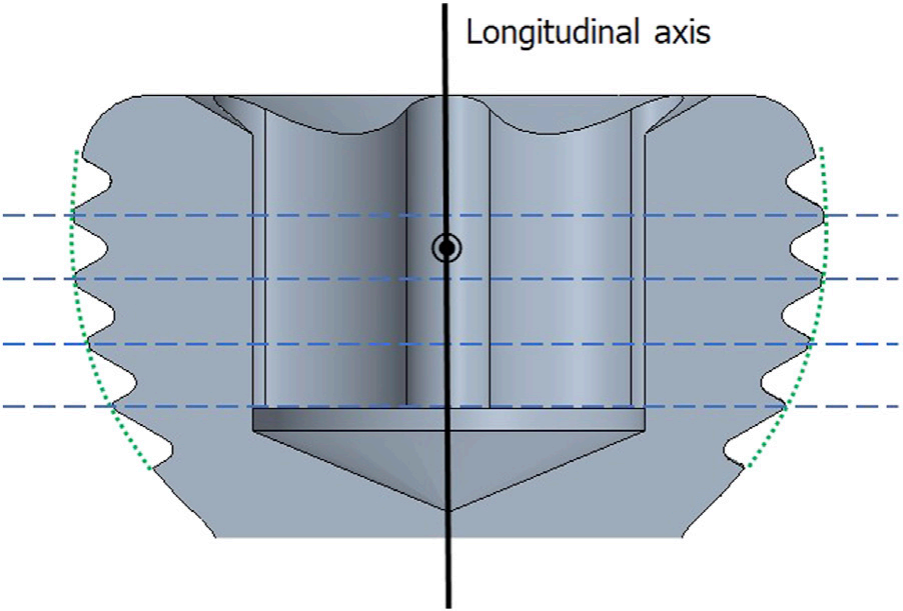
Schematic view of the screw head of the variable angle locking screw. *Green dotted curve* outlining the thread peaks represents a spherical-shaped screw head. Thread profile lines (b*lue dotted lines*) are parallel to each other and perpendicular to central axis.

**FIGURE 5:**
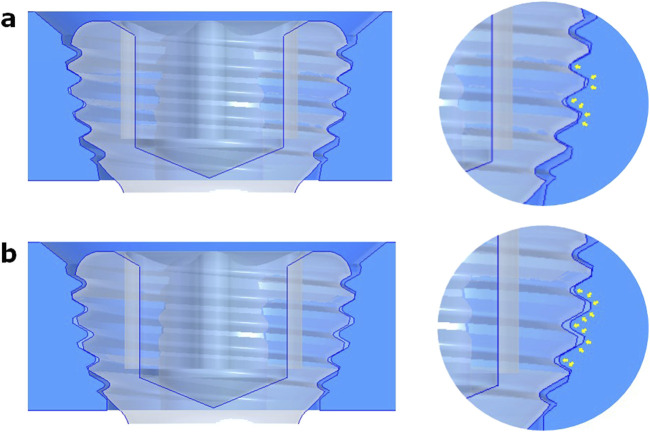
Schematic view illustrating the variable-angle locking screw (*in grey*) engaging with threaded holes (*in blue*) designed with **(a)** 20° and **(b)** 22° cone shapes. A magnified view shows the portions of cross-thread engagement (*yellow arrows*). The 22° conical hole has obviously more thread engagement than the 20° conical hole.

**FIGURE 6:**
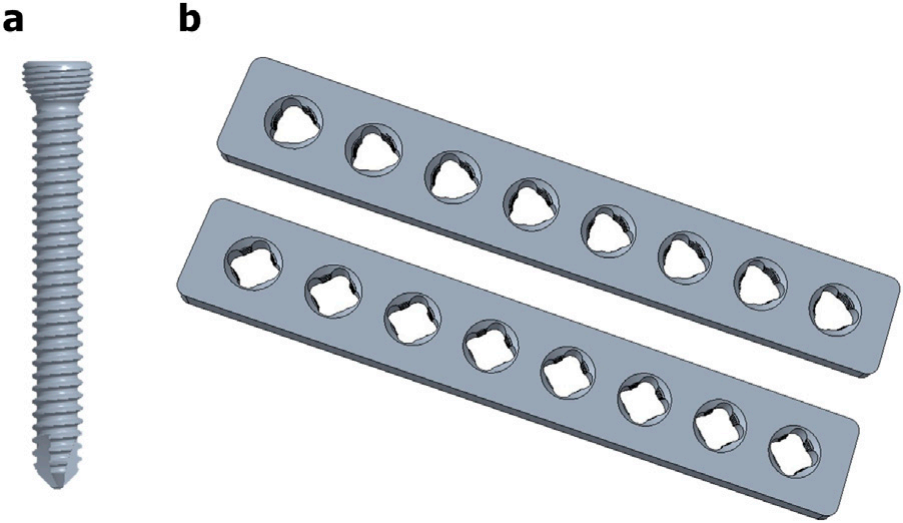
3D models of **(a)** the variable-angle locking screw and **(b)** the custom-made plates featuring three (upper) and four (lower) thread columns.

Three or four slots, forming corresponding thread columns (Figure 2), were proposed for the VALS hole design in this study. Mechanically, these slots are intended to facilitate cross-thread engagement between the screw head and the plate hole, thereby accommodating multidirectional screw insertion. Consequently, the performance of the variable-angle locking system is closely associated with both the slot size and the conical angle of the threaded hole. In most of commercialized bone plates, the conical angle of the threaded hole is 20° to accommodate both fixed-angle locking screw and variable-angle locking screw (Figure 3). For the feasibility of head thread fabrication of the variable-angle locking screw, the longitudinal axis of thread cutting bit is set perpendicular to longitudinal axis of the screw shaft, thus representing a manner that head thread profile lines is parallel to each other (Figure 4). Therefore, thread engagement between the plate hole and the screw head and the thread-to-thread contact area of the locking interface varies with the conical thread profile of the plate hole. Theoretically, a greater inclination of the hole profile results in more thread engagement (Figure 5). We proposed a 22° conical hole to be included in this study. Further, slot size of 1.3 and 1.5 mm was designed VALS hole for comparison. Therefore, 8 VALS hole features were applied in the custom-made plates for investigation (Table 1). 2.4 mm variable angle locking screws of 20 mm length and made of titanium (Depuy Synthes, Oberdorf, Switzerland) (Young’s modulus = 110 GPa; Poisson ratio = 0.3) were inserted into the VALS hole of titanium custom-made plates (Young’s modulus = 105 GPa; Poisson ratio = 0.37) (Figure 6). The holes of the custom-made plates were machined with the eight VALS design features. The thread pitch of the plate hole was designed at 0.3 mm to correspond to the head thread of the 2.4 mm variable angle locking screw. A guiding block was used to ensure the exact angle for the screw insertion into the VALS plate hole. Screws were inserted at different angles (0°, 5°, 10°, 15°) covering the full range of possible angulations of the VALS mechanism. All screws were tightened by 0.8 Nm torque.

**TABLE 1:** Variable angle locking hole features included in the test.

Thread column	Slot size/conical angle	Tested group
3 columns	1.3 mm/20°	V3-1320
3 columns	1.3 mm/22°	V3-1322
3 columns	1.5 mm/20°	V3-1520
3 columns	1.5 mm/22°	V3-1522
4 columns	1.3 mm/20°	V4-1320
4 columns	1.3 mm/22°	V4-1322
4 columns	1.5 mm/20°	V4-1520
4 columns	1.5 mm/22°	V4-1522

### 2.2 Biomechanical testing

**FIGURE 7:**
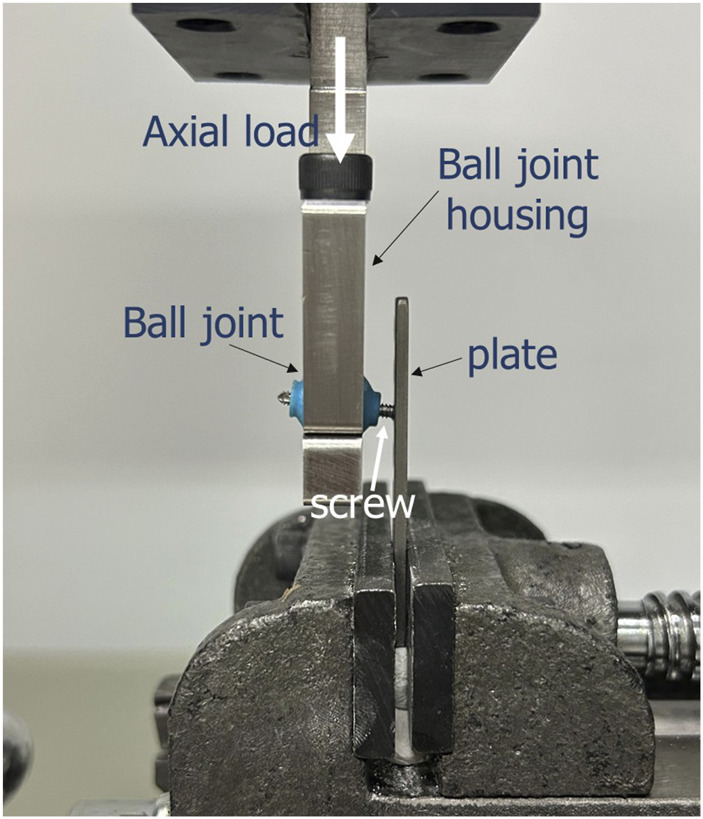
Diagram of biomechanical test setup. The custom plate was clamped by a vice. A vertical compression load was applied to the screw shaft at a distance of 4 mm from the plate.

Mechanical property investigation was conducted using a material testing machine (HT-2402, Hung-Ta Instrument Co., Taiwan) equipped with a 1000 N load cell, with the test setup illustrated in Figure 7. The VALS plate was securely fixed within a vise to allow for the application of axial compressive load. The screw shaft was inserted into the hole of a ball joint, which also functioned as a protective device. This is necessary to minimize point stress on the screw. As the screw specimen was loaded and displaced, the ball joint was able to rotate accordingly as the section moved downward (Collinge et al., 2023). The ball joint was connected via a linear slide to the actuator of the testing machine, which operated in displacement control mode at a speed of 1 mm/min. An axial load was applied at a distance of 4 mm from the undersurface of the plate. The test was continued until screw failure occurred, defined as (1) breakage, (2) a rapid force drop exceeding 50%, or (3) a displacement reaching 2 mm (Hebert-Davies et al., 2013). The axial displacement (mm) and load (N) were recorded from the actuator. The maximum load was determined from the load-displacement curve. Ultimate bending moment at the VALS interface was derived from multiplying the maximum load with the lever arm.

### 2.3 Statistical analysis

SPSS 21.0 (IBM Corporation, Armonk, NY, United States) was used for statistical analysis. Any significant differences between the four VALS groups were assessed using one-way analyses of variance. Post hoc analysis was conducted using Tukey multiple comparison test. The statistical significance was set at *p* < 0.05. Five tests were conducted per group for each angle.

## 3 Results

**FIGURE 8:**
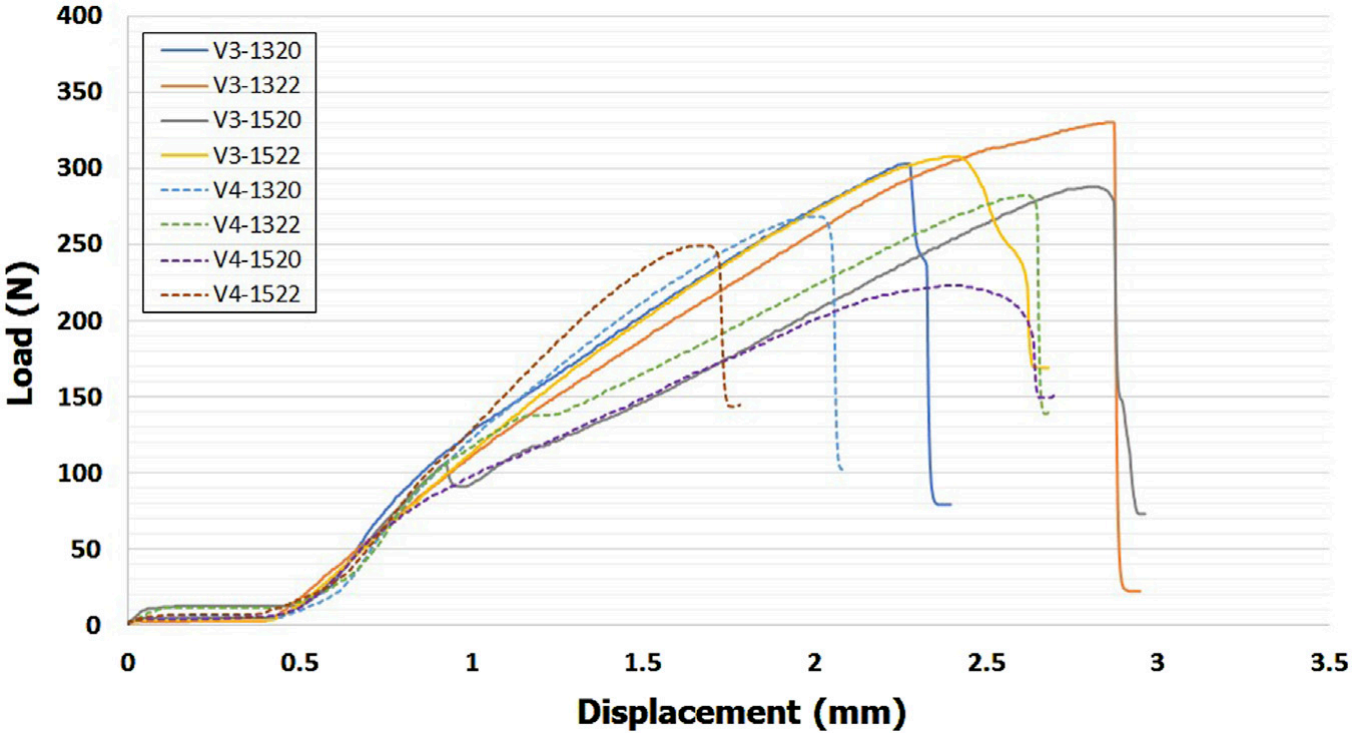
Load-displacement curves of the eight VALS designs at 10° screw insertion angle.

**FIGURE 9:**
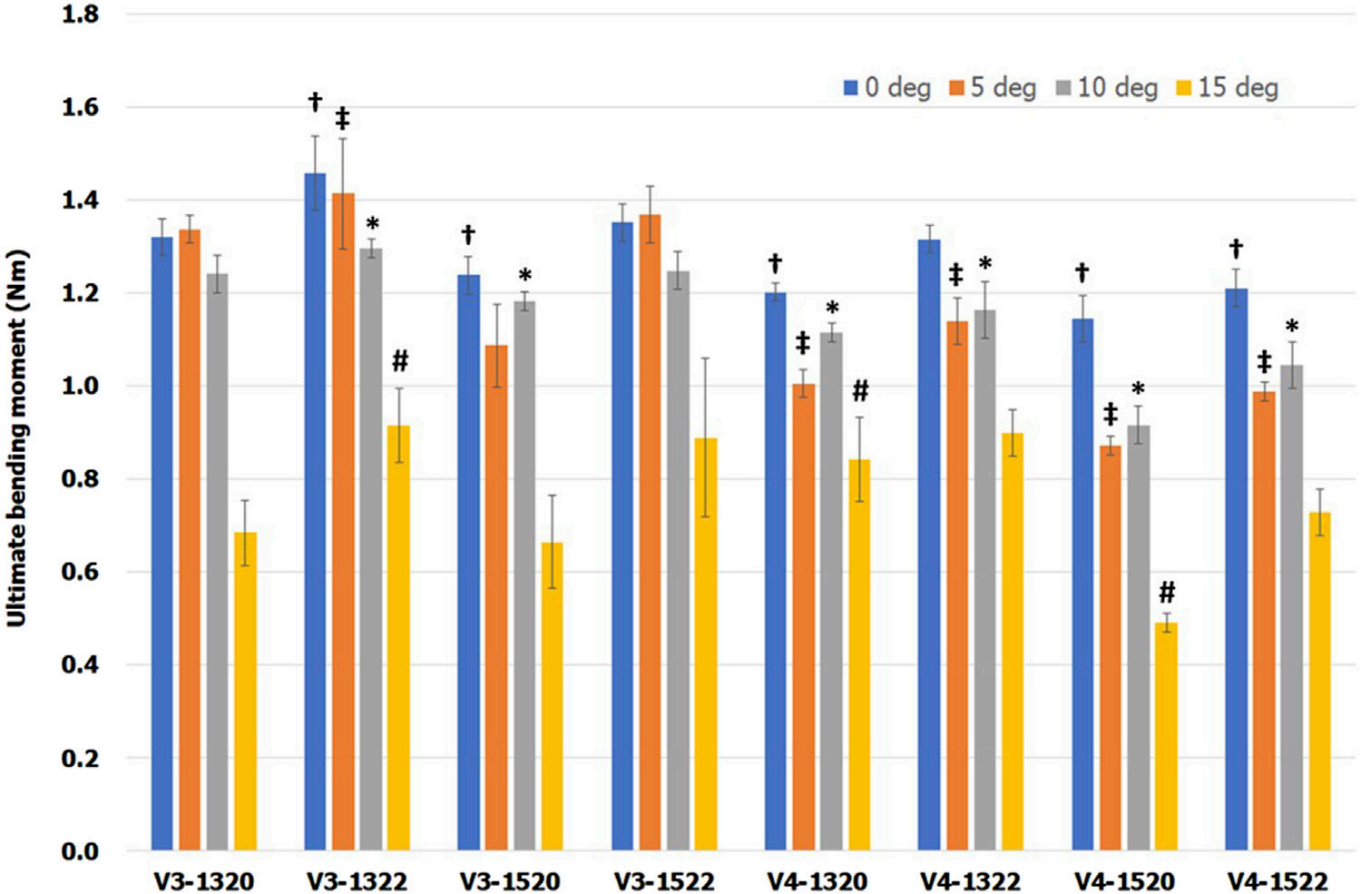
Mean ultimate bending moment with one standard error. Special markers (†, ‡, ✱ and #) indicate significant differences between VALS groups with the same insertion angle.

**FIGURE 10:**
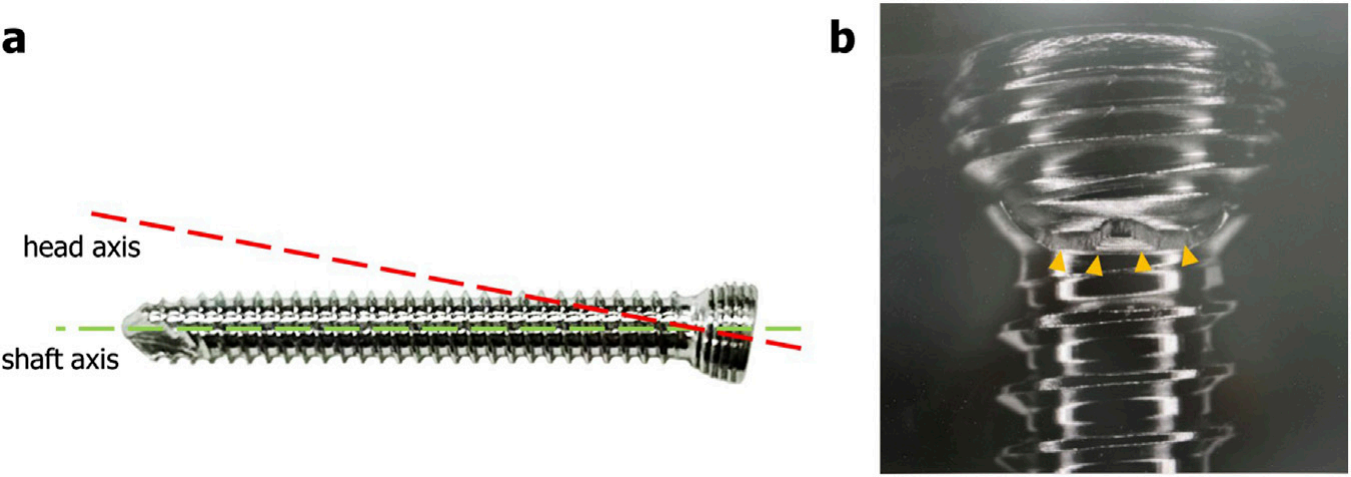
**(a)** Screw with catastrophic deformation at head-neck junction. **(b)** Screw breakage at head-neck junction indicated by arrows.

Typical force–displacement curves was shown in Figure 8. Mean ultimate bending moments for eight VALS holes at the different screw angulation is presented in Table 2 and a bar chart with statistical comparison results is shown in Figure 9. For all eight VALS groups, comparable bending moments were seen between off-axis insertions ranging from 0° to 10° though it slight decreased at both 5° and 10°. Ultimate bending moments decreased at a 15° inclination, with all 15° angulated VALS groups showing statistically significant differences compared to the 0° groups and all other off-axis VALS groups. The V3-1322 design showed higher bending moment than 7 other VALS designs at each insertion angles. Significant differences were seen at 0°–15°, with V3-1322 design being mechanically superior than V4-1320 (*p* < 0.05) and V4-1520 (*p* < 0.01) designs. The V3-1322 design also showed significantly higher moment at 5° and 10° off-axis compared to V4-1322 (*p* < 0.05) and V4-1522 (*p* < 0.01) designs. At 0°–10° inclination, all VALS locking screws failed due to breakage or plastic deformation at the neck region, while the screw heads remained fixed to the plate (Figure 10a). All screws showed macroscopic damage on the head thread (Figure 10b).

**TABLE 2:** Mean bending strength (Mean ± SD, Nm) in eight VALS hole designs at different screw insertion angles.

VALS group	0 deg	5 deg	10 deg	15 deg
V3-1320	1.32 ± 0.04	1.34 ± 0.03	1.24 ± 0.04	0.68 ± 0.07
V3-1322	1.46 ± 0.08	1.41 ± 0.12	1.30 ± 0.02	0.92 ± 0.08
V3-1520	1.24 ± 0.04	1.09 ± 0.06	1.18 ± 0.02	0.66 ± 0.10
V3-1522	1.35 ± 0.04	1.37 ± 0.09	1.25 ± 0.04	0.89 ± 0.17
V4-1320	1.20 ± 0.02	1.00 ± 0.03	1.11 ± 0.02	0.84 ± 0.09
V4-1322	1.32 ± 0.03	1.14 ± 0.05	1.16 ± 0.06	0.90 ± 0.05
V4-1520	1.15 ± 0.05	0.87 ± 0.02	0.92 ± 0.04	0.49 ± 0.02
V4-1522	1.21 ± 0.04	0.99 ± 0.02	1.05 ± 0.05	0.73 ± 0.05

In design models with identical thread columns and slot diameter, 22° conical hole exhibited greater strength than 20° cone design. Similarly, models with a smaller slot size (1.3 mm) or three thread columns (V3), with other features held constant, demonstrated higher load resistance compared to those with a larger slot size (1.5 mm) or thread columns (V4).

## 4 Discussion

Variable angle locking screws have been developed to overcome specific limitations associated with conventional fixed-angle locking systems, particularly in scenarios requiring more surgical flexibility. These screws enable enhanced freedom in screw trajectory by incorporating design mechanisms that facilitate secure locking in a non-perpendicular fashion, thereby accommodating complex anatomical structures and variable fracture patterns. Several studies on variable-angle plating systems have employed biomechanical testing using fracture models or full constructs with multiple screws to replicate clinical applications (Mehling et al., 2013; Hart et al., 2015; Nourbakhsh et al., 2019; Martínez-Fortún et al., 2024; Hoffmeier et al., 2009; Adler et al., 2017). Others have focused on the single screw–plate interface to isolate mechanical behavior (Hebert-Davies et al., 2013; Glowacki et al., 2023a; Lenz et al., 2015; Glowacki et al., 2023b). However, direct comparisons of bending moments across systems must be approached with caution, as differences in testing methods, plate designs, and locking mechanisms can significantly influence outcomes. Little is known how the variable angle hole profiles impact the capacity of the locking strength under different screw insertion directions. This study investigated the biomechanical performance of three distinct plate hole features under off-axis screw insertion conditions.

Our data revealed that small slotted design is advantageous for the locking strength of the variable-angle mechanism. This is reasonable because the plate hole with small slot size provides more thread-to-thread contact area at the locking interface. In the aspect of mechanics, better thread engagement would ensure better resistance against external load. Also, three slotted design groups represented superior bending strength than four slotted groups owing to the same rationale as mentioned above. Recently, Glowacki et al. used a micro-CT to examined the contact area between the variable angle locking interfaces (Glowacki et al., 2023b). The results demonstrated a strong correlation between bending strength and the thread engagement, which can support our finding.

**FIGURE 11:**
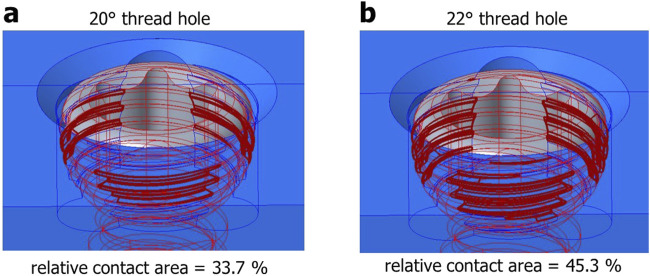
Visualization of the thread contact area in the variable-angle locking interface: **(a)** 20° conical hole; **(b)** 22° conical hole.

Furthermore, the conical design of the plate hole affected the VALS strength as well. As our hypothesis, 22° conical hole design has larger thread contact area than 20° conical hole design (Figure 11). Our results revealed that the 22° cone-shaped plate hole has higher bending moment than that of 20° conical hole. This was attributed to the conical geometry of the plate hole and the thread profile of screw head.

From a manufacturing perspective, the proposed slot size, cone angle, and thread configuration are technically feasible with modern machining but may increase production complexity and cost compared with conventional designs. These practical challenges should be considered alongside the biomechanical advantages when evaluating the clinical translation of the design.

Overall, our results showed that bending strength decreased as screw insertion angle increased, either in three-thread-column groups or in four-thread-column groups. The highest bending moment was 1.46 Nm among three-thread-column designs at 0° inclination while it was 1.32 Nm among four-thread-column designs. At 15° screw angulation, the highest moments were 0.92 Nm and 0.90 Nm for the three-thread and four-thread designs, respectively. In comparison, Lenz et al. simulated a variable angle locking hole design with four thread columns and 2.4 mm variable angle locking screws (Ti6Al7Nb alloy) (Lenz et al., 2015). They reported a statistically significant reduction at 15° compared to 2°–10°. Specifically, the bending moments were 1.67 Nm and 1.33 Nm at 0° and 15°, respectively. While the overall trend in bending moment was comparable, the magnitudes differed between our study and that of Lenz et al. These differences may be attributed to variations in experimental jigs, setup and the custom manufacturing of plates (such as the slot size and thread). Additionally, the material properties differed: our plates were pure titanium (yield strength = 483 MPa; tensile strength = 550 MPa), whereas Lenz et al. used Ti6Al7Nb alloy (yield strength = 800 MPa; tensile strength = 900 MPa). The higher mechanical strength of Ti6Al7Nb likely enhanced thread engagement at the screw–plate interface, which may partly explain the higher bending moments observed in their study.

Inserted at 15° inclination, all screws revealed a lower bending moment in the current study. Numerous studies also concluded similar finding (Hebert-Davies et al., 2013; Lenz et al., 2015; Glowacki et al., 2023b). In a cantilever load study, Hebert-Davies et al. (2013) comparing three commercialized VALS devices, found significant reduction of failure load of up to 45%. Lenz et al. (2015) revealed that difference in ultimate failure moment was statistically significant between the 15° angulated group and other off-axis groups. Recently, Glowacki et al. (2023b) tested the mechanical performance of three variable angle locking systems. They described a reduction of bending force of up to 57% with 15° of angulation. Accordingly, we suggested extreme off-axis of 15° should be used with caution and should not be exceeded.

This study focused on the investigation of design variable in point-loading thread-in mechanism. Several biomechanical evaluations have demonstrated this mechanism provides higher locking strength than the cut-in mechanism which utilizes a harder screw head to cut a track into a softer plate thread (Mehling et al., 2013; Hoffmeier et al., 2009; Adler et al., 2017). Consistent with related work (Lenz et al., 2015; Glowacki et al., 2023b; Adler et al., 2017), our results revealed a significant reduction in locking strength at screw inclination of 10°–15°. In contrast, Mehling et al. presented that the cut-in mechanism had the highest ultimate strength at large screw angulation (10°–15°) (Mehling et al., 2013). Similarly, Herbert-Davies et al. found that the locking cap mechanism tended to sustain higher loading at both 10° and 15° (Hebert-Davies et al., 2013). These differences may be attributed to the distinct contact mechanisms at the screw–plate interface.

This study contains several limitations:

1. The plate-screw head locking interfaces were tested under a cantilever load scenario which cannot reflect the physiological conditions.
2. We only focused on the locking interface rather than the whole plate-screw construct. While this mechanical study shows that all screws lose force when angulated, the clinical impact is uncertain. Further tests that apply the superior VALS design in the bone plate is necessary to evaluate the biomechanical properties in different simulated fracture patterns.
3. Screw loosening is a continuous process driven by cyclic loading. Although static loading tests in this study can serve as predictors of cyclic performance, fatigue testing is essential to further evaluate the biomechanical behavior of the better combination of plate hole design variables prior to clinical application.
4. A formal *a priori* power analysis was not conducted due to the limited availability of specimens and the large number of experimental groups. As a result, certain subgroup comparisons may be underpowered. Nevertheless, the primary objective of this study was to explore overall trends in plate hole design, rather than to establish definitive conclusions for each subgroup.
5. This study was limited to experimental testing. Future work employing finite element modeling or digital image correlation could provide more detailed validation of the contact mechanics.

## 5 Conclusion

This study demonstrated that the slot diameter, number of slots, and conical angle of the plate hole significantly influence the mechanical performance of the variable-angle locking interface under different screw angulations. A plate hole design variables—specifically a three slots, a 1.3 mm slot diameter, and a 22° conical angle—may provide improved mechanical performance under static loading conditions. Further efforts must be taken to find out the fatigue performance and clinical practice of a variable-angle locking plate system designed with these parameters.

## Data Availability

The original contributions presented in the study are included in the article/supplementary material, further inquiries can be directed to the corresponding author.
